# Indian Famine and English Hospitals

**Published:** 1897-01-23

**Authors:** 


### INDIAN FAMINE AND ENGLISH HOSPITALS.

A Mansion House Fund is so great a British institution, and the charity-compelling force exercised by the Lord Mayor when he gives his public sanction to any object and guarantees its worthiness is so enormous, that it need not be wondered at that those interested in other good objects look on with a certain amount of dismay when they see the stream of charity diverted into new and strange fields. There is too much reason for believing that, be the object what it may, the givers are practically the same. If a new appeal for the relief of a new form of distress assailed the pockets of a new and untouched class of contributors; if, while the hearts of some men were touched by the wants of the sick poor at our own doors, the hearts of others could only be melted and their purse-strings unloosed by the wretchedness and distress of our starving Indian fellow-subjects, every new form of appeal would be doing a double good; not only would it be relieving a larger area of distress, but it would be bringing fresh recruits into the ranks of the givers, and drawing many to interest themselves in the cause of charity who had hitherto been wrapped in selfishness and indifference to the woes of others.

We fear, however, that this is not so. It is true that certain individuals and certain firms, specially interested in our Indian trade, may give to help a Famine Fund who would not be influenced in any special degree by appeals for London hospitals; but in regard to a great deal of the money which goes to make up the sum—£100,000—which has already been given in response to the Lord Mayor's appeal, it is to be feared that it is largely a diversion of the stream of charity rather than an increase of its bulk. We have received letters from the secretaries of hospitals telling us that already the difficulty is being felt, that already the call of India is being given as a reason for declining to give towards the wants of London, and that in regard to certain City companies the money which is being sent abroad is really what, but for this appeal, would in all probability have come to the aid of distress nearer home. By all means let the citizens of London give mightily, let them pour out their wealth for the relief of the distress in India, distress which is sufficiently appalling. But let it be a new gift; let it be an addition to their usual offerings for the relief of misery and distress. Do not let us find, as we fear will be the case, that what is given to the new appeal is taken from the old, that it is a case of relieving the East at the expense of the West, of robbing Peter to pay Paul. It is not only our habit, but it is our duty, to urge the claims of our English hospitals, and much as we, in common with so many others, are moved by the call of India, we must nail to the mast the motto, "England first," and when we see, as at the present time we do see, reason to fear that the English sick, and the hospitals established for their relief, are likely to suffer in consequence of the popularity of another claim on the sympathies of the charitable, we cannot but call attention to the immensity of good which £100,000 would do at the present time if offered to the hospitals of London. £100,000 is a good, round, substantial sum, from the use of which the contributors may fairly wish to see some definite result. But in feeding the distressed millions of India it is but a drop in the bucket. If the Indian famine should assume the proportions which by some it is feared it will do, the real responsibility of feeding the people must of necessity always lie upon the Government, and thus all voluntary contributions will be a mere relief to the taxpayer, lessening by so much the sum he would have to pay himself if it were not paid for him. How different is the case of the home charities which we specially represent! Depending absolutely on voluntary contributions, trusting entirely from year to year to that fickle thing, reputation, which in turn depends upon the goodness of the work done day by day in full view of an ever critical public, our hospitals stand in constant want both of the gifts and of the sympathy of the charitable. We urge, then, that when people give to India they should give what otherwise they would not give at all. But England first. We would not see our English hospitals crippled for the relief, ostensibly, of India, but really for the relief of the Indian Government.

